# Development of Diopside-Modified Marl-Based Dielectric Composite for Microelectronics Applications

**DOI:** 10.3390/nano15090668

**Published:** 2025-04-27

**Authors:** Nassima Riouchi, Oussama Riouchi, Othmane Lamrani, El Hassan Yahakoub, Mohammed Mansori, Boštjan Genorio, Mitja Kolar, Petranka Petrova, Soufian El Barkany, Mohamed Abou-Salama, Mohamed Loutou

**Affiliations:** 1 LCM2E, Laboratory of Molecular Chemistry, Materials and Environment, Multidisciplinary Faculty of Nador (FPN), Mohammed Premier University, B.P. 300, Selouane, Nador 62700, Morocco; oussama.riouchi@ump.ac.ma (O.R.); s.elbarkany@ump.ac.ma (S.E.B.); m.abousalama@ump.ac.ma (M.A.-S.); m.loutou@ump.ac.ma (M.L.); 2 Laboratory of Geosciences, Environment Associated Resources, Faculty of Sciences Dhar ElMahraz, Sidi Mohamed Ben Abdellah University, Fez 30000, Morocco; othmane.lamrani@usmba.ac.ma; 3 LCME, Laboratory of Materials and Environmental Chemistry, Faculté des Sciences et Techniques, Université Cadi Ayyad, Av. A. Khattabi, BP 549, Marrakech 40000, Morocco; m.mansori@uca.ac.ma; 4 Faculty of Chemistry and Chemical Technology, University of Ljubljana, Večna pot 113, 1000 Ljubljana, Slovenia; bostjan.genorio@fkkt.uni-lj.si (B.G.); mitja.kolar@fkkt.uni-lj.si (M.K.); 5 Faculty of Mathematics and Natural Sciences, South-West University, Ivan Mihailov 66, 2700 Blagoevgrad, Bulgaria; ppd@swu.bg

**Keywords:** marl, diopside, composite material, dielectric properties, electronic applications, sustainable development

## Abstract

This research explores the modification of marl by the incorporation of diopside (CaMgSi_2_O_6_) to develop a composite material with improved dielectric properties, while addressing environmental and economic challenges through the use of abundant natural resources. X-ray fluorescence (XRF) analysis reveals a high silicate content in the raw marl, mainly SiO_2_ (68.12%) and Al_2_O_3_ (12.54%), while laser particle size analysis indicates a homogeneous grain size distribution centered around 100 µm. The composite was synthesized by the solid-state reaction method, achieving good phase homogeneity. X-ray diffraction (XRD) and infrared spectroscopy confirm the incorporation of diopside, while SEM analysis shows a porous morphology with granular aggregates. The modified material has an average particle size of 11.653 µm, optimizing the electrical properties. Impedance spectroscopy demonstrates improved dielectric performance, with accumulated permittivity and reduced losses, which improves energy storage and dissipation. Tests showed the remarkable stability of dielectric properties over a wide frequency range (10 Hz to 10 MHz) and low-temperature dependence. The performance was demonstrated on a single sample with a thickness of 0.63 mm, demonstrating consistent efficiency. These results position the diopside-modified marl as a promising candidate for electrochemical and microelectronic applications.

## 1. Introduction

Marl is a sedimentary rock composed mainly of clay, calcium carbonate (CaCO_3_) and sand [1]. It results from the accumulation of fine sediments in aquatic environments, such as seabeds, lakes or lagoons, where it is formed by the precipitation of calcium carbonate and the contribution of clay and siliceous particles [2]. This varied composition gives marl unique physicochemical properties that allow it to be exploited in various industrial fields [3].

Widely used in the construction industry, marl is used in particular as a raw material in the manufacture of cement, where it is included in the composition of mixtures intended for the production of clinker [4]. Its calcium carbonate content plays an essential role in the firing reaction of cement, while the clay fraction provides the silica and alumina necessary for the formation of the mineral phases of the clinker [5]. In addition, marl is used in the production of bricks, tiles and ceramics, where it improves the plasticity of the mixtures and contributes to the mechanical resistance of the finished products after firing [6].

Outside the construction sector, marl also finds applications in agriculture, where it is used as a limestone amendment to correct soil acidity and improve its structure [7]. It is sometimes added to substrate formulations to optimize water retention and nutrient availability for plants. In some chemical industries, marl can also be exploited as a mineral filler in the manufacture of paints, plastics and cosmic products [8].

In the context of this study, marl and diopside should be identified as materials connected to emerging technologies and materials aimed at playing a role in sustainability, particularly in the context of the green economy and waste management [9]. The exploitation of these resources can be part of an approach to optimizing transformation processes to reduce their environmental impact and promote the circular economy.

Diopside is a mineral belonging to the pyroxene group, composed mainly of calcium and magnesium silicate (CaMgSi_2_O_6_) [10]. It is particularly appreciated for its physical and chemical properties, including its thermal stability, mechanical strength and electrical characteristics [11]. When associated with marl, diopside can significantly improve the performance of the latter by modifying its structural and functional properties, in particular by optimizing its permittivity and, in particular, dielectric losses.

The incorporation of diopside into a marl matrix thus makes it possible to obtain materials with improved performance for advanced applications in the fields of electronics and electrical engineering. Due to its low electrical conductivity and its ability to withstand high temperatures, diopside reinforces the stability of composite materials and limits the effects of energy dissipation under electrical stress [12]. These characteristics are particularly sought after in the manufacture of capacitors, high-frequency insulators and components for power transmission systems [13].

Future research should further investigate the impact of different strategies to improve the electrical performance of dielectric materials. Among these approaches, those developed by Carlos, E. et al. [14] highlight an innovative technique combining solution combustion synthesis and UV irradiation. This method has several potential advantages, including improved homogeneity of the resulting films, reduced synthesis temperature and optimized structural and electrical properties of the materials. A detailed analysis of this approach will provide a better understanding of the interactions between the microstructure of dielectric films, their crystallinity and their electrical response as a function of synthesis parameters and post-deposition processing conditions.

Furthermore, the relationship between the permittivity of materials and their band gaps represents a fundamental challenge for the development of new high-performance materials. As indicated by the work of Barquinha, P. et al. [15], precise control of this interaction is essential to optimize the energy efficiency of electronic devices and limit dielectric losses. The ability of a material to confine charges and minimize leakage currents depends closely on the match between its permittivity range and its band gap. Therefore, it is necessary to conduct in-depth studies on different types of dielectrics, exploring combinations of oxides, nitrides and hybrid materials in order to identify the most suitable structures for applications in microelectronics, optoelectronics and advanced telecommunications.

Optimizing dielectric films for electronic applications requires a thorough understanding of their structural and dimensional properties, including their compactness and thickness. The density and porosity of films directly influence their electrical performance, affecting charge permeability and device stability. A structure that is too porous can lead to leakage currents and impair insulation, while a material that is too dense may cause adhesion and mechanical stress problems. Similarly, film thickness plays a key role in the operation of microelectronic components, as too little thickness promotes tunneling effects and reduces dielectric strength, while excessive thickness can limit miniaturization and increase power consumption.

To better control these parameters, advanced characterization techniques are essential, such as impedance spectroscopy for electrical analysis, SEM and AFM for morphology and roughness and RBS and ellipsometry to measure thickness and density. These analyses allow us to optimize the conditions of synthesis and the integration of films in next-generation electronic devices. These advances will also contribute to the improvement of materials for advanced telecommunications systems, where stability, reliability and low electrical conductivity are essential criteria to minimize energy losses and ensure high performance.

## 2. Materials and Methods

### 2.1. Types of Materials

**Figure 1:**
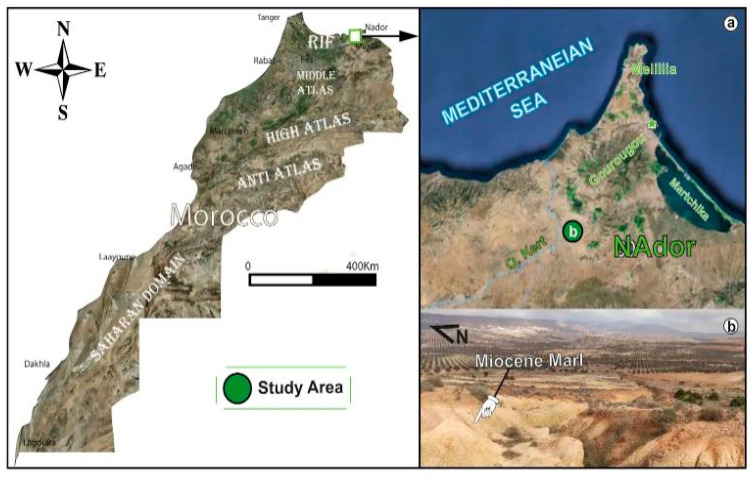
Geographical and geological location of the Miocene marl mining area near Nador, Morocco, with a detailed map of the coastal region (**a**) and a view of the mining site near a dam (**b**).

Figure 1 presents the geographic location of the marl extraction area near Nador in northeastern Morocco. The map on the left shows the main geological formations of the country, including the Rif, Middle Atlas, High Atlas and Anti-Atlas mountains. The study area is marked in green in the Nador region near the Mediterranean Sea. The map on the right provides a close-up of this area with details of the coastal region around Nador, including natural features such as the Oued Kert and localities like Melilla. Inset (b) presents a view of the extraction site where Miocene marl visible in the landscape was collected near a local dam for scientific study.

### 2.2. Experimental Procedures

#### Procedures

This study began with the preparation of marl using an innovative solid-state modification method with diopside. To transform this raw material, the modification was carried out by incorporating calcium oxide (CaO) and magnesium oxide (MgO) powders, both with a purity of 99%. These powders were carefully weighed and ground for 30 min to obtain a homogeneous mixture. The mixture was then subjected to a sintering process at 1000 °C for three hours with a controlled temperature increase of 5 °C per minute. In parallel, we studied various parameters influencing the sintering process, such as temperature, particle size and granulation methods. For this, we applied several physicochemical characterization techniques, including X-ray diffraction (XRD). Fourier-transform infrared spectroscopy (FTIR) scanning electron microscopy was coupled with energy-dispersive X-ray analysis (SEM-EDX) and dielectric analysis. These approaches enhanced our understanding of the material synthesis process and deepened our knowledge of their properties.

### 2.3. Characterization Methods

To characterize our samples we employed a range of analytical techniques. X-ray diffraction (XRD) was used to examine the post-sintering crystalline structure. This analysis was performed using a Bruker D8 diffractometer operating at 40 kV and 40 mA. The scan covered a 2θ angle range from 10° to 80° at a speed of 2.4°/min with a CuKα wavelength of 1.5406 Å. The data obtained were processed with JCPDS software (now part of the ICDD database, such as PDF-5+ 2025 and JADE Pro 2025) to identify crystalline phases, and the crystallite size (hkl) was determined using the Debye–Scherrer formula.

To measure the density of the sintered samples we used the Archimedes method with a Mettler Toledo ML204 balance. Hardness and toughness values were obtained by measuring indentation and crack dimensions with an average calculated over 10 measurements.

Elemental analysis was conducted using X-ray fluorescence spectrometry (XRF) which utilized an X-ray tube or radioisotope source to generate high-energy X-rays detected to determine the sample composition.

Laser granulometry relies on the diffraction of a laser beam through a particle suspension allowing particle size measurement. The angles and intensity of the diffracted light were analyzed using mathematical models to determine the particle size distribution. This method is fast precise and suitable for various industries to assess material quality.

Field-emission scanning electron microscopy (FE-SEM) (FEI Quanta 250, Hillsboro, OR, USA) with an EDS detector (Oxford 150 mm^2^, Abingdon, UK) was used to examine raw nano-powders from the sonicated mixture and fracture surfaces of sintered samples. The samples were coated with a thin layer of platinum using a Quorum Q150R coater (Laughton, East Sussex, UK), and nanoparticle dimensions were measured with ImageJ^®^ software.

FT-IR spectroscopy measurements were performed with a Bruker spectrometer using high-purity KBr, and spectra were recorded in the 4000–400 cm^−1^ range to analyze material vibrations at room temperature.

Finally for impedance spectroscopy we implemented an experimental method to analyze system responses to different electrical signals. This involved sample preparation impedance spectroscopy setup application of a low-amplitude sinusoidal electrical signal with gradual frequency variation and simultaneous measurement of voltage and current at each frequency. This approach allows for the calculation of the system’s complex impedance decomposed into a real part (resistance) and an imaginary part (reactance).

## 3. Results and Discussions

### 3.1. Mineralogy and Granulometric Properties of Raw Marl

#### 3.1.1. X-Ray Fluorescence

Table 1 presents the X-ray fluorescence (XRF) analysis, which aims to determine the precise chemical composition of the raw marl to identify its constituent elements and evaluate its properties for potential modifications or industrial applications. According to the results, the raw marl contains various elements, but is primarily composed of silicate materials with a high presence of SiO_2_ (68.12%) and Al_2_O_3_ (12.54%). These two components, silica and alumina, are typical of clay and silicate materials. Their significant presence indicates that the marl is rich in silicate minerals, characteristic of clay-based geological formations. This information is crucial, as it helps to assess the potential of marl for specific applications, particularly in the cement industry or for chemical modifications. The fact that the marl is predominantly silicate-based suggests it could be transformed to enhance certain properties as needed, for example, to increase its mechanical, thermal or dielectric capacities.

#### 3.1.2. Laser Granulometry

**Figure 2:**
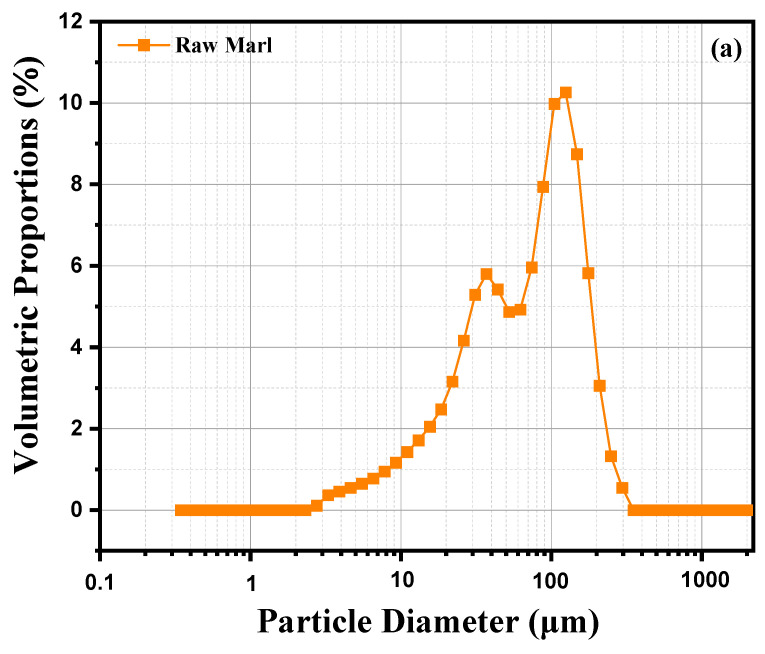

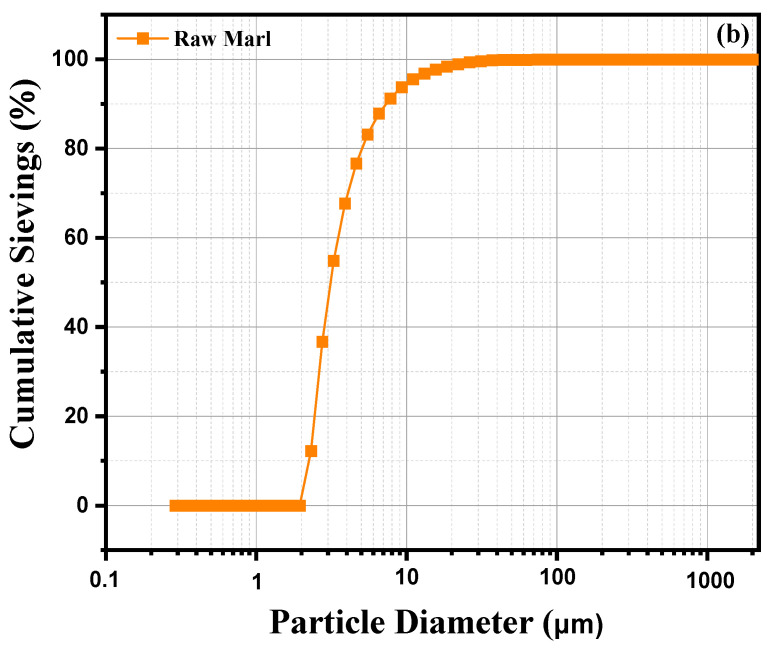
Laser Granulometry of raw marl: (**a**) volumetric percentage vs. particle size; (**b**) cumulative granulometric distribution vs. particle diameter.

This analysis examines the particle size distribution of a sample of marl clay, based on a graph illustrating the volume percentage distribution of the different particle sizes. Curve 2 (a) shows a pronounced peak around 100 µm, indicating that the majority of particles in this marl clay sample have a diameter close to this value, suggesting a significant concentration of medium-sized particles. In addition to this main peak, we observe a small proportion of fine particles (<10 µm), as well as a few larger particles (>100 µm). This distribution reveals a relatively homogeneous particle size composition characterized by a predominance of medium-sized particles and a smaller quantity of very fine or very coarse particles. The grain size is typical of marly materials, which generally contain a mixture of fine to medium particles. The preponderance of particles around 100 µm may indicate a stable depositional environment where particles of this size are favored. Finer particles could indicate phases of weathering or slow erosion, while larger particles could result from episodic inputs or more energetic transport conditions. This diversity in particle sizes reflects the complexity of the formation and deposition processes of marl clay. Figure 2b represents the cumulative particle size distribution of raw marl as a function of particle diameter obtained by sieving. The horizontal x-axis shows particle diameter in micrometers (μm) on a logarithmic scale, while the vertical y-axis shows the cumulative percentage of particles passing through a sieve of given diameter. A percentage of 100% means that all particles passed, while a percentage of 0% indicates that no particles of that size passed. The curve reveals a rapid increase in cumulative sieving percentage as particle size increases. This sigmoid shape indicates a relatively narrow particle size distribution, suggesting a majority of particles of similar sizes. The curve begins to rise markedly around at 1 μm, suggesting that the majority of particles have a diameter greater than this value. Around 10 μm, almost all of the particles have passed, with a cumulative sieving close to 100%. This indicates that a significant proportion of particles have a diameter between 1 and 10 μm. Beyond 100 μm, almost 100% of the particles passed through the sieve, showing that few particles of this size or larger are present. This distribution suggests that raw marl is composed mainly of fine particles, which are often sought after in applications such as the manufacture of cement or other construction materials. Particle fineness can also influence the chemical reactivity of marl, a crucial factor in certain industrial processes. Fine particles can provide good cohesion in mixes, but may also require specific treatments to avoid excessive compaction or water retention.

### 3.2. Mineralogy and Microstructure Changes

#### 3.2.1. X-Ray Diffraction Analysis

**Figure 3:**
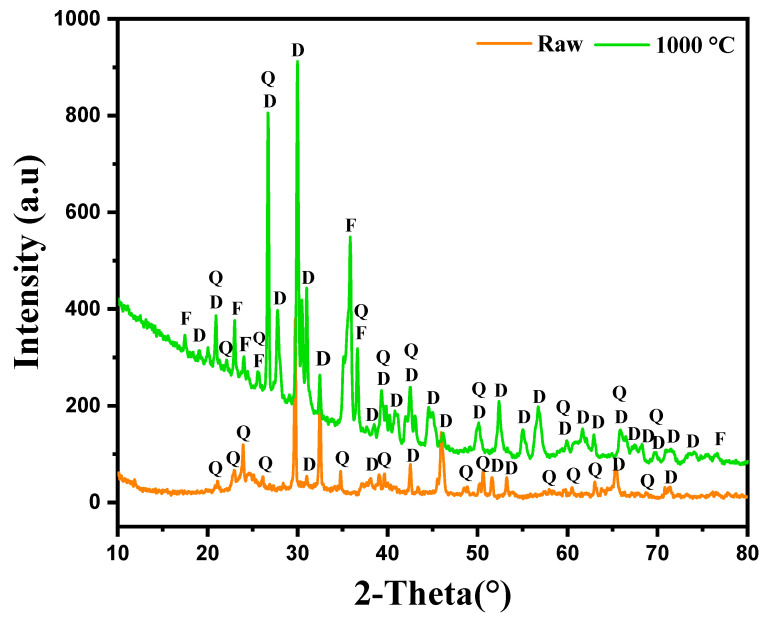
X-ray diffraction of marl samples modified with diopsidediopside in the raw state and after calcination at 1000 °C.

The XRD analysis conducted on the marl modified with diopside, both in its raw state and after calcination at 1000 °C, highlighted the presence of various mineral phases, their evolution and changes in peak positions after heat treatment, as illustrated in Figure 3. In the raw sample, the XRD results reveal the presence of two main phases: Quartz (Q) and diopside (D). The characteristic peaks of these phases are clearly visible on the diffractogram; Quartz, in a stable phase at room temperature, shows its most intense peak around 2θ = 26.6°. Diopside, on the other hand, displays lower-intensity peaks, suggesting it is present but not yet fully crystallized, or in a lower proportion within the marl matrix. After calcination at 1000 °C, the diffractogram shows significant changes in the sample’s crystalline structure. The intensity of the peaks corresponding to diopside (D) increases markedly, indicating enhanced crystallization of this phase due to the effect of temperature. Quartz (Q), though still present, does not show a significant variation in peak intensity, suggesting that this phase remains stable post-calcination. An additional phenomenon observed after calcination is the shift of certain peaks toward lower angles, particularly for diopside. This shift may indicate changes in the crystal lattice parameters, likely related to thermal relaxation or changes in the chemical composition of the phases under the effect of temperature. Such shifts are often observed when elements diffuse or rearrange within the crystal lattice during thermal treatment. Furthermore, new peaks appear after calcination, corresponding to the formation of Forsterite (F). Forsterite, a magnesium-rich mineral phase, generally forms at high temperatures from magnesium and silicon compounds present in the marl. Its formation indicates significant thermal transformations at 1000 °C, favoring the emergence of this new crystalline phase.

#### 3.2.2. IR Spectroscopy

**Figure 4:**
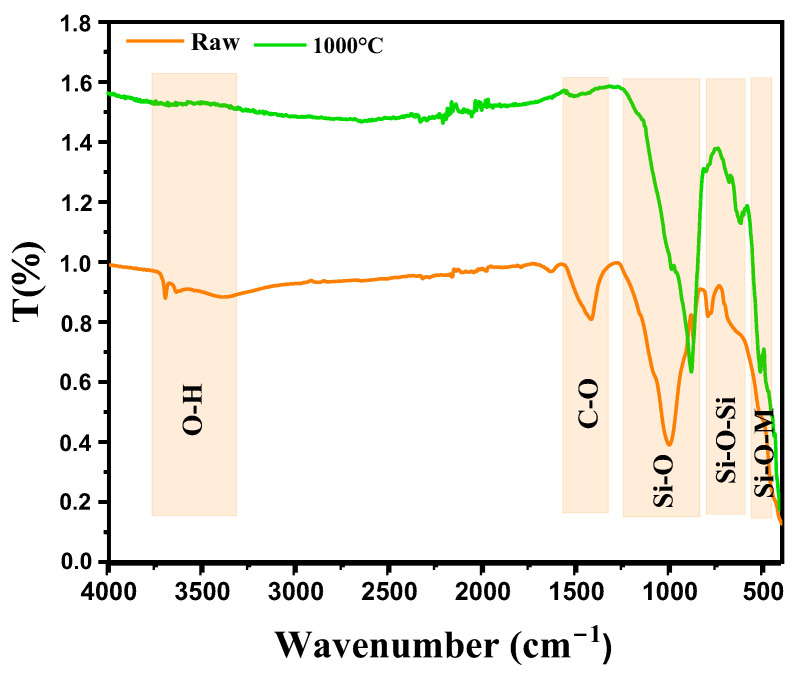
IR spectra of diopside-modified marl samples in their raw state and after heat treatment at 1000 °C.

Figure 4 shows the analysis of the infrared (IR) bands observed in diopside-based marl (CaMgSi_2_O_6_), both in its raw state and after thermal treatment at 1000 °C, revealing significant structural changes. In the raw state, the band at 3444 cm^−1^ likely corresponds to O-H vibrations from hydroxyl groups or adsorbed water, suggesting residual moisture or hydroxyls typical in unheated clay or marl materials [16]. The band at 1425 cm^−1^ is generally associated with carbonate (C-O) group vibrations, often as impurities in marl, indicating residual carbonates like CaCO_3_ that have not yet decomposed [17]. The band at 999.17 cm^−1^ is attributed to asymmetric stretching vibrations of Si-O bonds within the silicate network of the diopside structure, reflecting the SiO_4_ tetrahedral structure of the marl’s silicate phase. The band at 792.33 cm^−1^ corresponds to Si-O-Si vibrations in silicate networks, typical of silicates like diopside, representing interactions between SiO_4_ tetrahedra in the material structure [18,19]. After thermal treatment at 1000 °C, new bands appear, indicating structural modifications. The band at 881 cm^−1^ could be related to increased crystallization or reorganization of the silicate network, possibly indicating a more stable phase or reorganization of the SiO_4_ tetrahedra typical of diopside transformation after heating. The band at 621 cm^−1^, corresponding to Si-O deformations within silicate networks, suggests structural modifications with increased crystallization and improved thermal stabilization of diopside [20]. The band at 512 cm^−1^ is associated with bending vibrations of Si-O-M bonds (M being a metal such as Ca or Mg) within the crystal network, becoming more pronounced in post-thermal treatment, indicating a crystalline reorganization and better alignment of metal ions with the SiO_4_ tetrahedra [21].

#### 3.2.3. Scanning Electron Microscopy (SEM)

**Figure 5:**
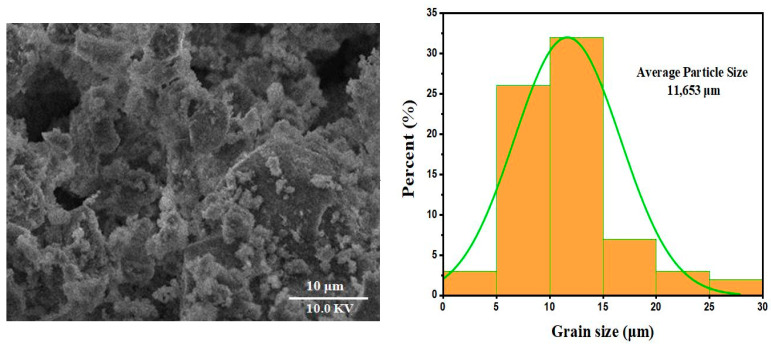
SEM micrograph and granulometric distribution study of samples sintered at 1000 °C.

The image obtained by scanning electron microscopy (SEM) provides a morphological analysis of marl modified by the addition of diopside. At a scale of 10 µm, several notable features can be observed, as illustrated in Figure 5. The granulometry was measured using ImageJ^®^ software (version 1.48e) with a size of 11.653 µm, offering insights into the structure and potential properties of the resulting material. First, the image reveals a porous morphology marked by the presence of cavities and channels. This porosity may indicate a significant transformation of the marl following modification with diopside. Such a porous structure is often sought after in fields like electrochemistry or catalysis, where increased active surface area can enhance reaction performance. Next, granular aggregates are observed, likely linked to the presence of diopside in the form of clusters within the marl matrix. This phenomenon could result from the interaction between the two materials during the modification stages, potentially through thermal or sintering processes. The presence of these aggregates suggests an uneven distribution of diopside, which may influence the physical and chemical properties of the composite material. Finally, the irregular surface structure observed in the image may be the result of thermal and mechanical stresses experienced during the preparation of the material. These irregularities could affect the overall mechanical properties of the composite, as well as its dielectric properties. Such heterogeneity in the structure may also impact its ability to conduct or store energy, which is crucial in microelectronic and electrochemical applications.

### 3.3. Dielectric Properties

#### 3.3.1. Study of Dielectric Properties

Impedance spectroscopy is a commonly used electrochemical analysis technique to characterize material properties in response to an alternating electrical excitation (AC) over a wide range of frequencies. It enables the analysis of impedance, which is composed of a real part (resistance) and an imaginary part (reactance), providing detailed information about conduction, polarization and relaxation processes within the materials [22]. During measurement, four parameters are recorded: the real and imaginary parts of the impedance, the phase angle and the dielectric loss. Additionally, the complex permittivity (ε*) is expressed in terms of its real (ε′) and imaginary (ε″) components, offering a better understanding of the electrochemical properties.

$$\varepsilon^{*}=\varepsilon^{\prime }-j\varepsilon^{\prime \prime }\left|\varepsilon^{*}\right|=\sqrt{{\left(\varepsilon^{\prime }\right)}^{2}+{\left(\varepsilon^{\prime \prime }\right)}^{2}}$$

**Figure 6:**
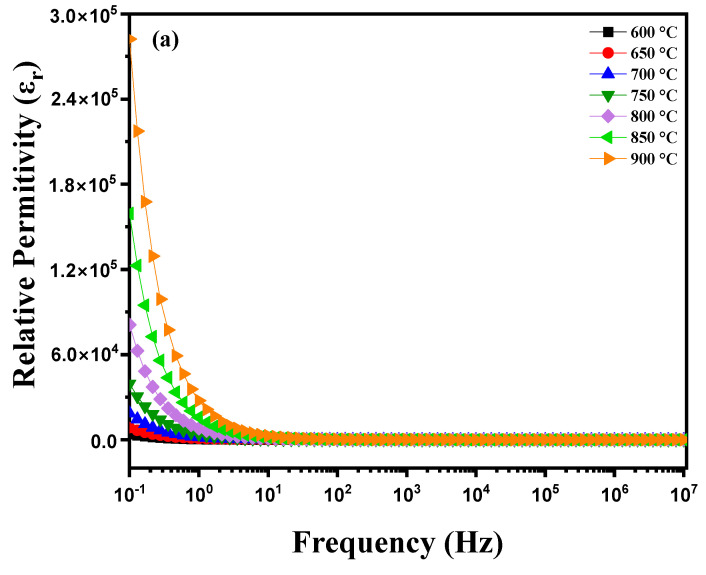

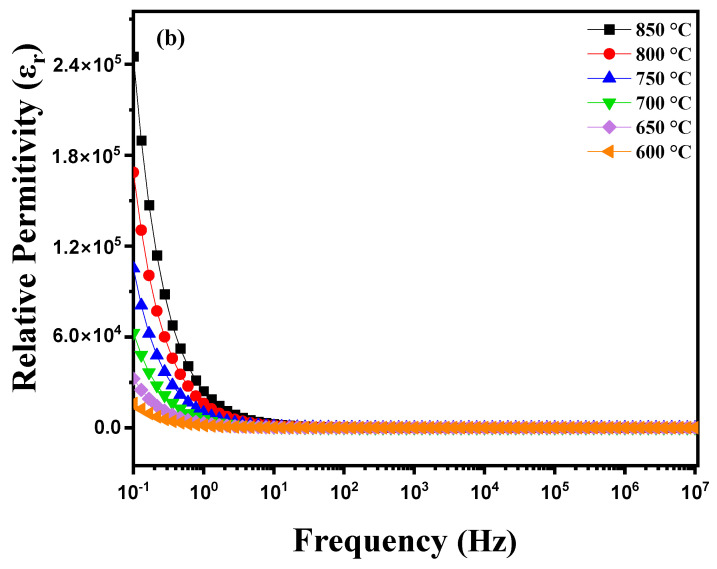
Diagram of relative permittivity variation as a function of temperature: (**a**) heating curve from 600 °C to 900 °C and (**b**) cooling curve from 850 °C to 600 °C for modified marl.

The values of relative permittivity (dielectric constant εr) and dielectric losses (tanδ) were calculated from the complex impedance data Z* (Z* = Z′ + jZ″) by applying the following equations. This type of analysis is commonly used to characterize dielectric materials and their electrochemical responses as a function of frequency and temperature. The relationships between permittivity, dielectric losses and complex impedance provide a better understanding of the conduction and relaxation mechanisms present in the material [23,24]:

(1) ε′=tωAε0×−Z″Z′2+Z″2;ε″=tωAε0×Z′Z′2+Z″2 tan⁡δ=ε″ε′

where ω = 2πf with f = frequency (Hz), A = pellet area (m^2^), t = pellet thickness (m), ε_0_ vacuum permittivity (ε_0_ = 8.85 10−12 F m^−1^), Z′= real part of the impedance and Z″ = imaginary part of the impedance. Figure 6a illustrates the variation in the dielectric constant (ε_r_) as a function of frequency for a diopside-modified marl disk measured at different temperatures between 600 °C and 900 °C. Several phenomena are observed: heating induces structural changes and atomic rearrangements within the material. As the temperature increases, atoms gain energy, which can cause phase transitions, crystallization of certain amorphous phases or ion diffusion. These modifications directly affect the dielectric properties of the material. The dielectric constant (ε_r_) is measured by applying an alternating electric field of various frequencies to the sample using an impedance analyzer. Electrodes placed on either side of the disk measure the material’s response to this field. The collected data show how ε_r_ varies with frequency at different temperatures. At low frequencies, contributions to permittivity are primarily due to interfacial polarization and ionic migration, while at high frequencies, dipolar and electronic polarization become predominant. Variations in ε_r_ with temperature reveal dielectric relaxation mechanisms and thermally activated phase transitions [25,26,27]. These insights are crucial for understanding how diopside modification affects the dielectric properties of marl, and how these properties evolve under the influence of temperature and frequency. Figure 6b shows the evolution of the dielectric constant (ε_r_) of this disk as a function of frequency at different temperatures during the cooling process. As the disk cools, significant changes may occur in its dielectric properties, reflecting the effect of decreasing temperature on the material. The diopside modification aims to enhance the electrical and mechanical characteristics of the marl, and these changes can be observed through variations in ε_r_ with frequency during the cooling process. At high temperatures, the molecules in the disk have high thermal energy, which facilitates their orientation under an applied electric field, thereby increasing the dielectric constant. In contrast, at low temperatures, thermal energy decreases, reducing the mobility of the molecules and their ability to orient, which can lead to a decrease in the dielectric constant. Furthermore, phase transitions may occur in the modified diopside, directly influencing the electrical properties of the material. Crystal structures can reorganize, causing variations in the dielectric constant. These transitions may be more pronounced at certain temperatures, leading to abrupt changes in εr values. Depending on the frequency of the applied electric field, dielectric losses may also vary. At low frequencies, dipoles have more time to align with the electric field, which can increase εr. At high frequencies, dipoles may not follow the field as effectively, reducing the dielectric constant.

**Figure 7:**
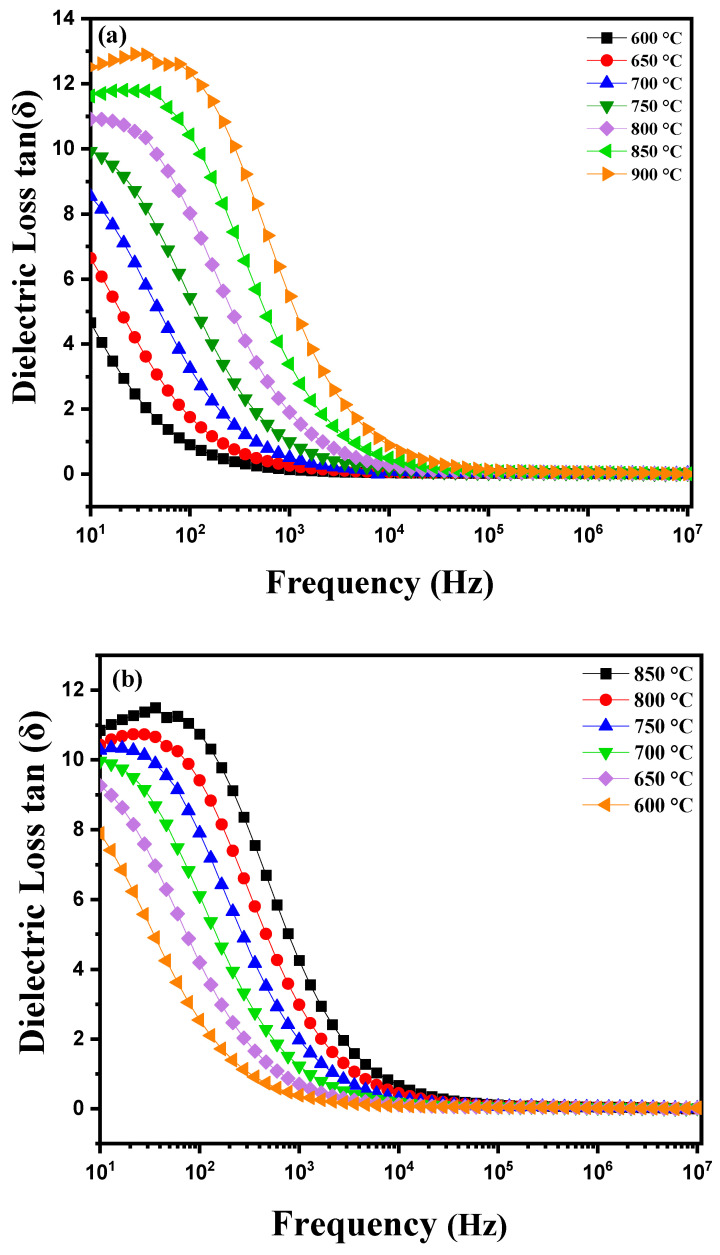
Diagram of dielectric loss variation as a function of temperature: (**a**) heating curve from 600 °C to 900 °C and (**b**) cooling curve from −850 °C to 600 °C for modified marl.

Figure 7a shows the dielectric losses (tanδ) as a function of frequency for a diopside-modified marl pellet during heating. As the temperature increases, several phenomena can occur. Diopside, a pyroxene with the chemical composition CaMgSi_2_O_6_, is known for its unique electrical and thermal properties. When heating this pellet, variations in dielectric losses can be observed, which often represent the energy dissipation in the material. The dielectric loss as a function of frequency is typically represented by a curve. At low frequencies, losses may be relatively high due to dipolar polarization and ionic conductivity. As the frequency increases, losses may decrease, reaching a minimum at a certain frequency before increasing again at higher frequencies due to conduction losses and dipolar relaxation [28,29]. The presence of thermally activated charge carriers is a crucial factor in this process. When the material is heated, charge carriers (electrons and holes) gain enough energy to overcome energy barriers and become mobile. This leads to an increase in the material’s electrical conductivity. At higher temperatures, these charge carriers can be more easily thermally activated, resulting in increased conductivity and, consequently, dielectric losses [30]. Figure 7b illustrates the variations in dielectric losses as a function of frequency during the cooling of this modified marl pellet. These variations reveal specific characteristics. Generally, at higher temperatures the charge carriers in the material (such as ions or electrons) are more active due to the increase in thermal energy. These charge carriers contribute to the material’s conductivity and influence dielectric losses. As the temperature decreases, the thermal energy available to activate charge carriers also decreases. This results in a decrease in the mobility of charge carriers and consequently a reduction in the material’s electrical conductivity. On a dielectric loss curve, as a function of frequency, this thermal transition may manifest as a noticeable decrease in dielectric losses at lower frequencies, where thermally activated charge carriers have a significant impact. At higher frequencies, dielectric losses may be dominated by other mechanisms such as dipolar polarization or intrinsic dielectric losses of the material. These mechanisms are less sensitive to temperature variations, which can lead to a relatively stable dielectric loss curve at high frequencies, even when the temperature decreases.

**Figure 8:**
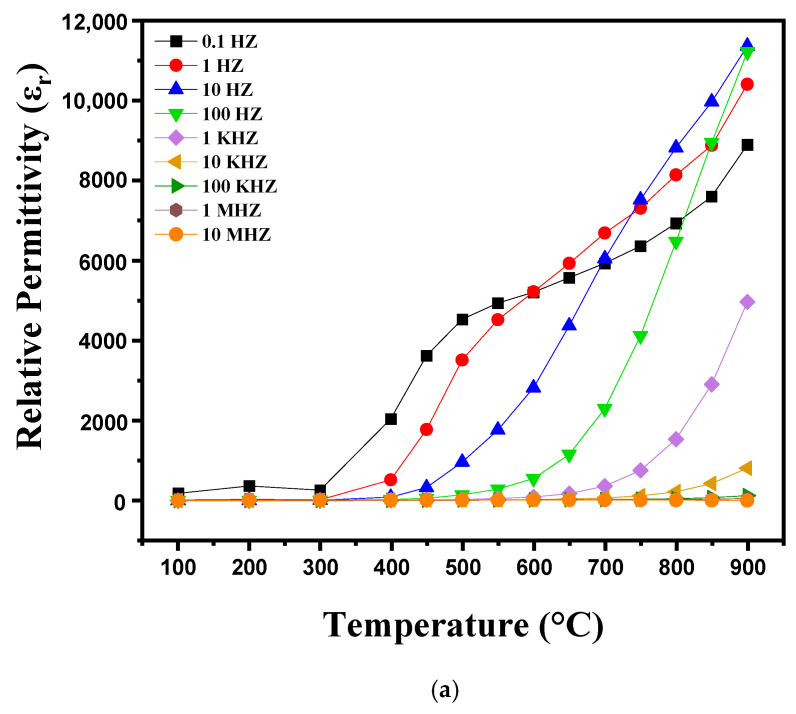

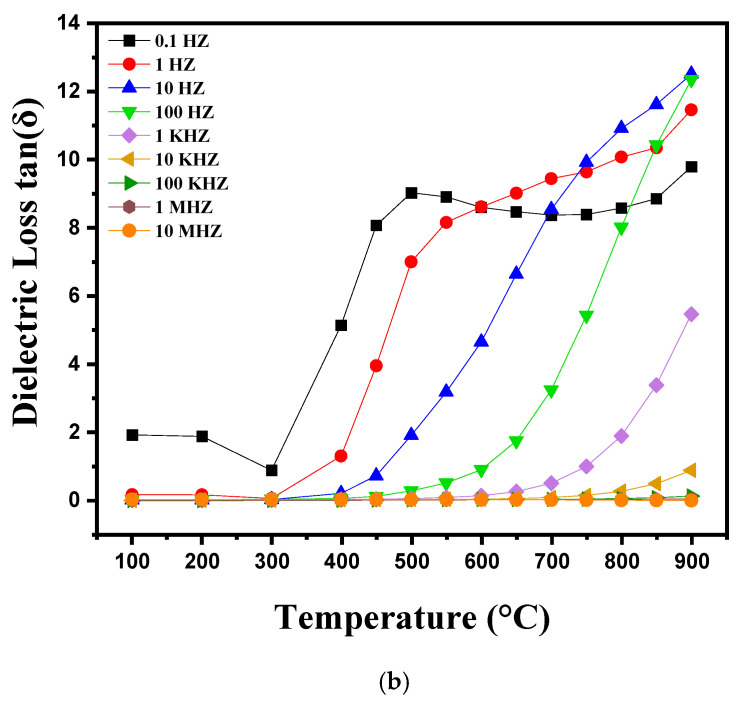
Diagram showing the evolution of (**a**) relative permittivity and (**b**) dielectric loss as a function of temperature, from 100 °C to 900 °C, as well as frequency variation.

Figure 8a,b shows the variations in dielectric losses and dielectric constant (ε_r_) as a function of temperature ranging from 100 °C to 900 °C at different frequencies (0.1 Hz. 1 Hz. 10 Hz. 100 Hz. 1 kHz. 10 kHz. 100 kHz. 1 MHz. 10 MHz). Distinct behaviors are observed in these variations. Between 100 °C and 300 °C, variations in the dielectric constant (ε_r_) and dielectric losses (tanδ) are relatively small and stable, which suggests that the polarization mechanisms remain stable and are barely influenced by frequency in this interval. The dipoles are partially misaligned and fail to align effectively under the electric field due to insufficient thermal energy at these temperatures. Thus, at this temperature, the dielectric properties initially decrease before showing a slight increase as thermal energy allows for better polarization. Above 300 °C, the dielectric constant and dielectric losses increase significantly with temperature. This behavior is attributed to temperature-dependent dipolar polarization, where the dipoles align more efficiently with the electric field as the thermal energy increases. At the same time, electrical conduction is amplified, which also increases εr and tanδ. Ceramics then undergo significant structural transitions, such as dehydration or changes in crystalline phases, which contribute to increased dielectric losses and increased dielectric constant. This phenomenon continues up to temperatures close to 900 °C, where the dielectric properties tend to stabilize or decrease slightly due to major transformations of the material [31,32].

#### 3.3.2. Complex Impedance Analysis (CIA)

**Figure 9:**
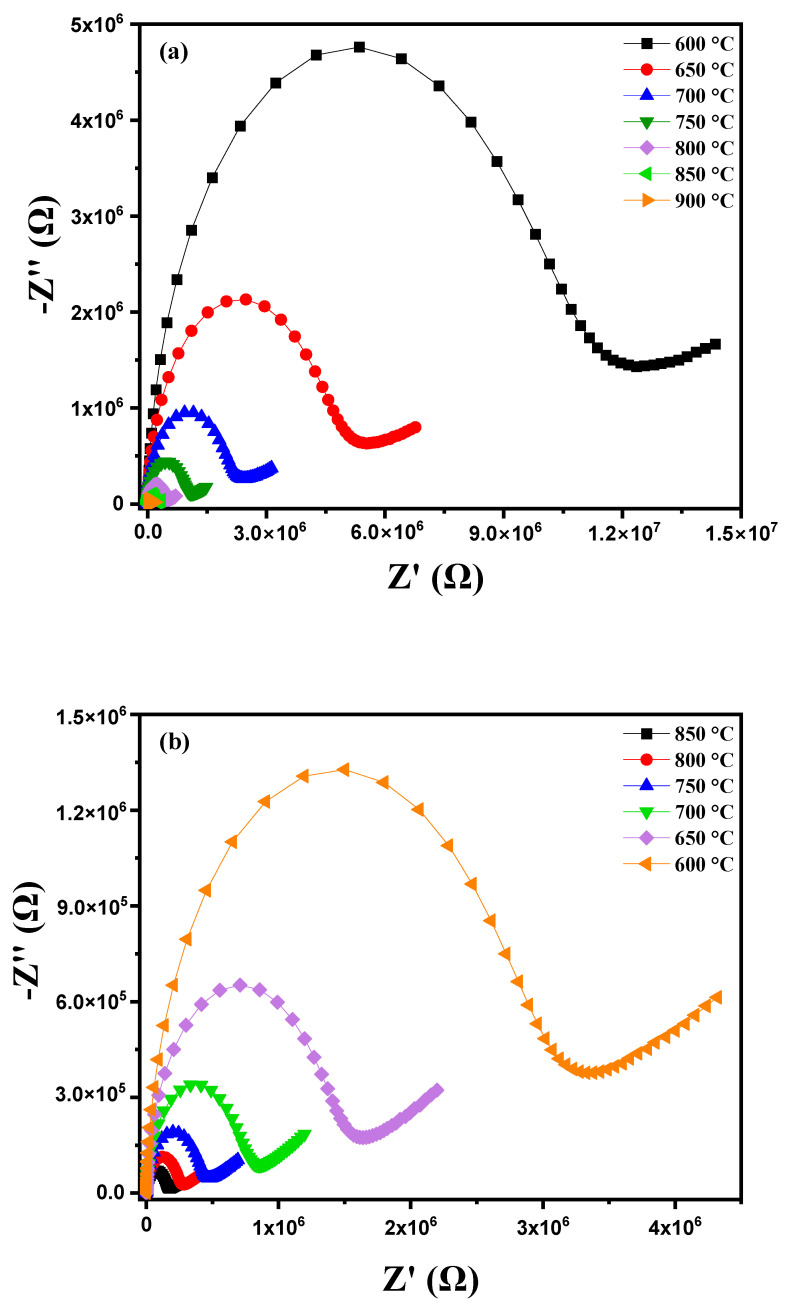

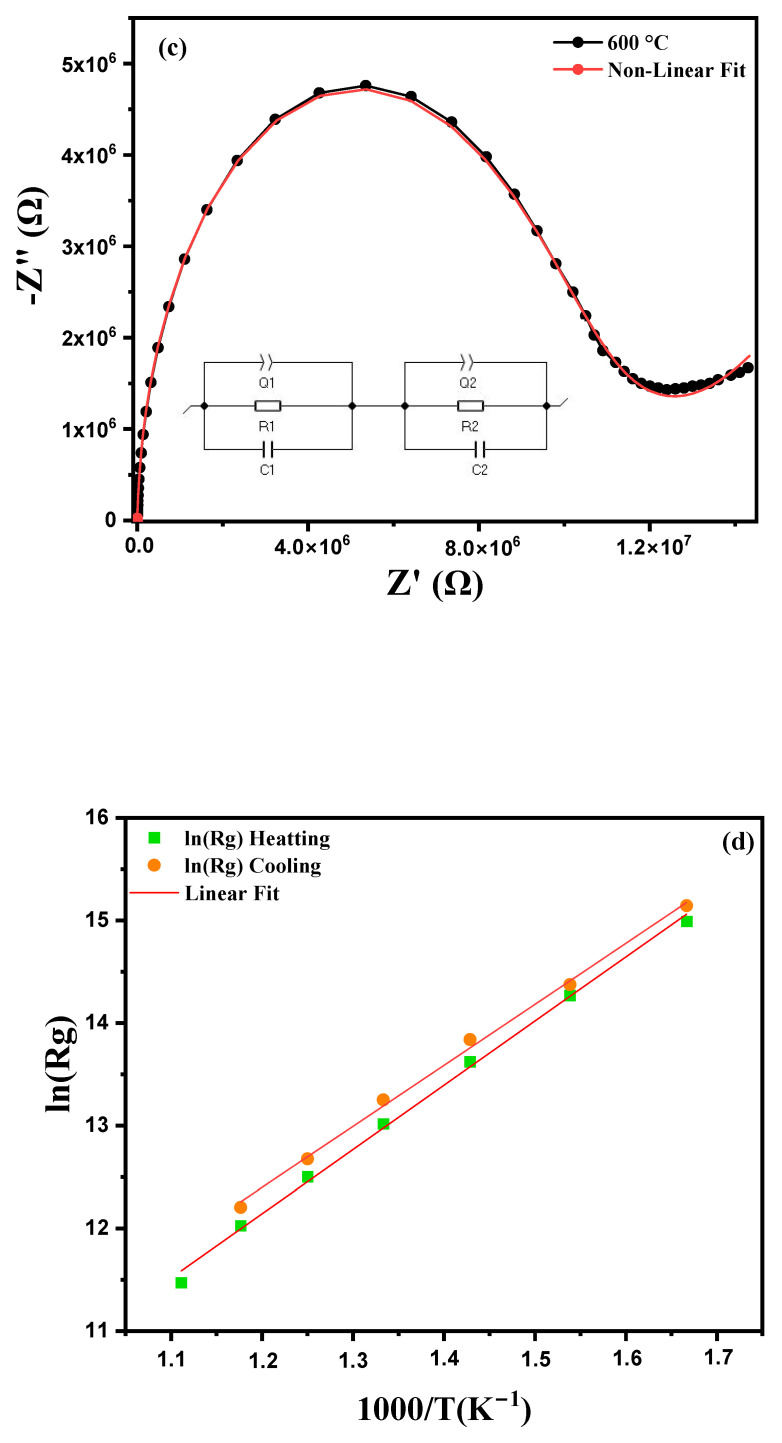

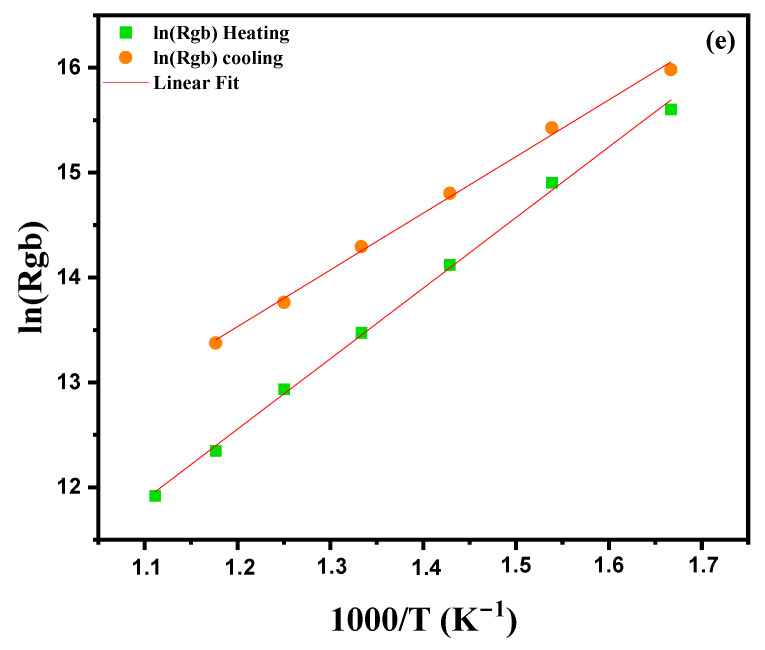
Cole–Cole plots for modified marl: (**a**) heating curve from 600 °C to 900 °C, (**b**) cooling curve, (**c**) equivalent circuit model at 600 °C, (**d**) Arrhenius plot for grain impedance and (**e**) Arrhenius plot for grain boundary impedance.

The complex impedance analysis is an advanced method using Nyquist plots to deeply explore the electrical properties of materials. This technique examines resistive, capacitive, inductive and reactive behaviors, highlighting the contributions of grains, grain boundaries and electrode effects. The Nyquist plots, where the imaginary part Z″ is plotted against the real part Z′, reveal arcs or semicircles indicating relaxation and conduction mechanisms, as well as polarization effects and contributions from interfaces. This analysis helps understand the distribution of relaxation times and complex conduction phenomena. The complex impedance Z* is determined by the following expression [33]:

Z* = Z′ + jZ″

where Z′ represents the real part of the impedance corresponding to the material’s ohmic resistance, Z″ represents the imaginary part of the impedance associated with reactance, which may be due to capacitive or inductive effects, and j is the imaginary unit $(j^{2}=-\sqrt{1}$). The formulas for Z′ and Z″ are as follows:

$$Z^{\prime }=\frac{R_{1}}{1+{\left(\omega R_{1}R_{1}\right)}^{2}}+\frac{R_{2}}{1+{\left(\omega R_{2}R_{2}\right)}^{2}}$$

$$Z^{”}=-\omega \left(\frac{R_{1}C_{1}}{1+{\left(\omega R_{1}C_{1}\right)}^{2}}+\frac{R_{2}C_{2}}{1+{\left(\omega R_{2}C_{2}\right)}^{2}}\right)$$

where R_1_, R_2_ and ω (with ω = 2πf) correspond with the grain resistance, grain boundary resistance and the angular frequency of the material, respectively. By plotting the Nyquist diagrams of the imaginary part against the real part of the impedance at different temperatures (600 °C to 900 °C), Figure 9a highlights several important phenomena. As the temperature increases, a reduction in the radius of the semicircles on the Nyquist diagrams is observed. This reduction in radius indicates a decrease in the material’s resistance, meaning that electrical conduction becomes more efficient [34,35]. This improvement in electrical conduction is due to the thermal activation of charge carriers. In other words, the increase in temperature provides the energy necessary to excite electrons and holes, thereby increasing their mobility. At higher temperatures, the increased mobility of charge carriers leads to a significant reduction in the material’s impedance. The semicircles thus become smaller, reflecting better conductivity [36]. This behavior indicates that the conduction process in the diopside-modified marl pellet is thermally activated. Heat facilitates the movement of charge carriers through the material, reducing the energy barriers that hinder their movement at lower temperatures. When heating a diopside-modified marl pellet, a reduction in the radius of the semicircles on the Nyquist diagrams is observed as the temperature increases. This indicates a decrease in the material’s resistance and an improvement in electrical conduction, demonstrating that the conduction process is thermally activated. These observations provide valuable insights into the electrochemical properties of the material and its potential applications in various technologies. Figure 9b shows that during the cooling of this pellet, the Nyquist diagram traced at different temperatures between 850 °C and 600 °C reveals several notable phenomena. As the temperature decreases, a progressive enlargement in the radius of the semicircles in the Nyquist diagram is observed. This indicates an increase in the material’s resistance to electrical conduction. Indeed, at lower temperatures, the mobility of charge carriers is reduced, making it more difficult for them to move through the material. The conduction process is thermally activated, meaning that the decrease in temperature reduces the available thermal energy to activate charge carriers, thus decreasing their mobility and increasing resistance.

In material studies, an equivalent circuit model is often used to represent the complex electrical properties of grains and grain boundaries. This circuit, expressed as Q1/R1/C1 + Q2/R2/C2 segments the electrical response of the material into various distinct components each capturing a particular aspect of the electrical behavior. Additionally, Z-View software (version 4.0) allows for the adjustment of the parameters of each element in this circuit, providing a precise optimization of the fit between experimental data and the model shown in Figure 9c. This model can be described by a series circuit consisting of two distinct branches. The cell Q1/R1/C1 models the response of the grain boundaries and the interfaces between the grains of the material. Q1: This is a constant phase element (CPE) used to model irregularities in charge distribution at the grain boundaries. R1 represents the resistance of the grain boundaries. Grain boundaries are often more resistive due to the presence of structural defects or impurities which increase the resistance to electrical conduction at these interfaces. C1 corresponds to the capacitance of the grain boundaries. This capacitance reflects the accumulation of charges at the grain interface, causing a capacitive effect due to the difficulty in transporting charges across the grain boundaries. The second branch of the circuit, Q2/R2/C2, describes the response of the grains in the material, which are generally more conductive than the grain boundaries. Q2: This constant phase element (CPE) models the slightly non-ideal electrochemical response within the grains. Although conduction is more homogeneous in the grains, minor imperfections justify the use of a CPE. R2 represents the internal resistance of the grains. Grains that are more conductive than the grain boundaries have a lower resistance, facilitating the conduction of charges through them. C2 corresponds to the capacitance within the grains [37]. This capacitance is related to charge polarization in the grains, but it is typically less pronounced than in the grain boundaries. The constant phase element (CPE) is used in grains and grain boundaries to model their non-ideal capacitive behavior. This non-idealism may result from the presence of several distinct relaxation processes [38,39]. The capacitance C and the value of the constant phase element (CPE) are linked by the following formula:

C = (R − Q^α^)^1/α^

R is the resistance, Q is the CPE parameter and α is the phase index (0 < α ≤ 1). The improved Nyquist diagram, as well as the equivalent circuits for the compound, are presented in Figure 9c. The values of R, C and CPE, associated with the contributions of grains and grain boundaries, MT-Lab software (version 6.0) was used. A good agreement between the experimental data and the calculated values confirms the validity of the proposed equivalent circuit. The different adjusted parameters are presented in Table 2, which shows that the resistances Rg (grain) and Rgb (grain boundaries) decrease with increasing temperature. This decrease in Rg and Rgb is linked to two factors: thermal activation and the release of trapped charge carriers with the increase in temperature, which confirms the semiconductor nature [40]. In addition, we observe that the resistance of the grains (Rg) is always lower than that of the grain boundaries (Rgb) for all the temperatures studied. The values of grain resistances (Rg) and grain boundaries (Rgb) obtained from the Nyquist plots in Figure 9a,b were analyzed using the following Arrhenius law to evaluate their activation energy [41]:

$$R=R_{0}exp(-Ea/k_{B}T)$$

where Ea is the activation energy for conduction, K_b_ is the Boltzmann constant, R_0_ is the pre-exponential factor and T is the temperature in kelvins. Comparison of grain strength (ln(Rg)) and grain boundary strength (ln(Rgb)) curves versus 1000/T during heating and cooling cycles reveals both similarities and notable differences. Curve 9 (d, e) shows that the grain boundary resistance (ln(Rgb)) is systematically higher than the grain resistance (ln(Rg)), with a marks hysteresis between the heating and cooling cycles. This hysteresis, characterized by steeper slopes during cooling, suggests the presence of irreversible phenomena, such as structural changes or thermal stresses. Furthermore, the resistances increase at low temperatures and decrease at high temperatures, indicating thermally activated behavior, as confirmed by several recent studies. These results highlight the importance of grain–grain interfaces and thermal effects on the electrical properties of the material. The values Eg (grain activation energy) and Egb (grain boundary activation energy), obtained by linear adjustment with this equation, show that the grain activation energies are at 0.469 eV during heating and at 0.555 eV during cooling, while those of grain boundaries vary between 0.647 eV during heating and 0.889 eV during cooling, for given temperatures. The higher values of grain boundary activation energies are probably related to the presence of structural defects, impurities or thermal stresses at the grain–grain interfaces. These defects and irregularities in the grain boundary structure increase the resistance to charge transport, which explains the increase in activation energy. Differences between heating and cooling values also result in irreversible phenomena or structural changes in grain boundaries during temperature variations.

#### 3.3.3. Conductivity Study

The electrical conductivity of a material is a measure of its ability to conduct an electric current. It depends on the availability and mobility of charge carriers (electrons or ions) through the material. Electrical conductivity can be calculated from the dielectric parameters using the following formula:

σ_AC_ = ε_0_ε_r_tan(δ)

where ε_0_ is the permittivity of free space, ε_r_ denotes the relative permittivity of the material and tan(δ) represents the dielectric loss factor. This expression links the AC conductivity σ_AC_ to the energy storage and dissipation properties of the material. When the AC conductivity is plotted as a function of angular frequency (ω) at different temperatures between 600 °C and 900 °C, the curve 10a reveals several distinct behaviors depending on the frequency and temperature regions. At low frequencies, the curve typically shows a plateau region, where the AC conductivity is relatively stable and corresponds to DC conduction. This region is characterized by the mobility of free charges such as ions and electrons which have enough time to move under the effect of the alternating electric field. At these temperatures, the increase in temperature improves conductivity in this region by facilitating the mobility of charges, resulting in a general increase in the plateau as the temperature rises from 600 °C to 900 °C. In the intermediate region of the curve, where conductivity shows a nonlinear variation, behaviors indicative of more complex conduction mechanisms are often observed. This region suggests a short-range hopping of charge carriers where charges move in steps or by local jumps rather than by continuous movement. At higher temperatures, this region may expand or shift, reflecting an improvement in the hopping conduction mechanisms as the charges gain more energy to overcome potential barriers. At high frequencies, the conductivity behavior is generally attributed to a process of localized or reoriented ion hopping. At these high frequencies, charges are less able to follow the rapid variations in the electric field, and conductivity is dominated by ion relaxation processes or dipole reorientation mechanisms. As the temperature increases, these processes may become more efficient, resulting in a more significant increase in conductivity at high frequencies. The conductivity curves at low temperatures are well described by Jonscher’s double power law, expressed as follows [42,43]:

σ(ω) = σ_DC_ + AωS^1^ + BωS^2^

In this Formula, (S1) and (S2) represent the frequency exponents for the medium- (104–106 Hz) and high-frequency (>106 Hz) regions, respectively, while A and B are the pre-exponential factors associated with conductivity. As the temperature increases, S1 tends toward 0, indicating the dominance of DC conductivity in the low-frequency range. The conductivity curves of the sample appear to follow Jonscher’s power law, which is given by [44].

σ_AC_ = σ_DC_ + AωS

**Figure 10:**
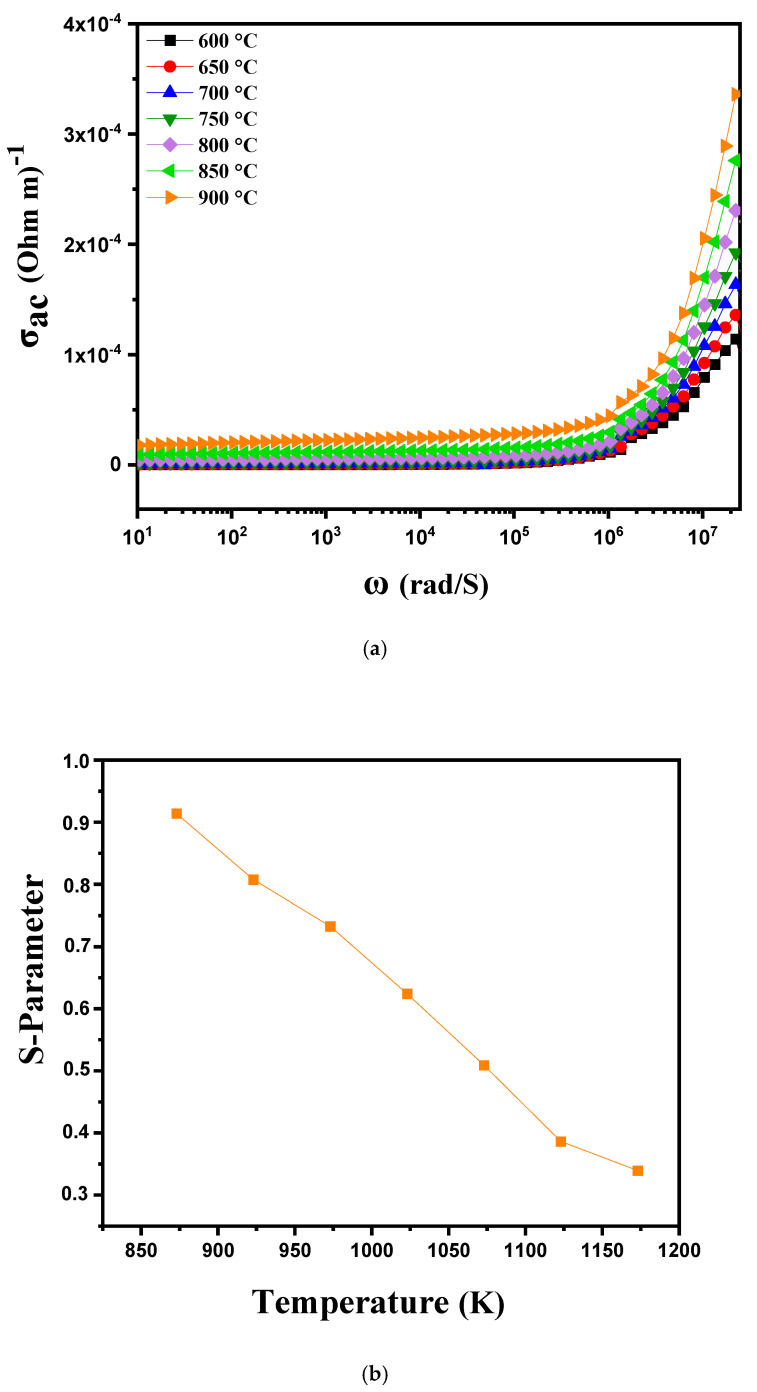
(**a**) AC conductivity as a function of frequency at different temperatures from 600 °C to 900 °C for modified marl and (**b**) variation in the “s” parameter with temperature.

In this expression, σ_DC_ denotes the direct current (DC) conductivity of the material, while A is the pre-exponential factor determining the extent of polarization. The value of S is essential for analyzing conduction mechanisms in materials and assessing the transport properties of charge carriers such as vacancies, electrons and ions [45]. When S is greater than 1 (S > 1), this indicates that charge carriers move by localized hopping. Conversely, when S is less than 1 (S < 1), charge carriers move via translation [38,46]. The temperature dependence of the S parameter, in the range 600 to 900 °C, is presented in Figure 10b, showing that “S” varies inversely with temperature. This confirms that the correlated barrier jumping (CBH) model is the most appropriate model to explain the charge transport mechanism in these samples [47]. According to this model, the AC conductivity in these samples results from the charge carriers jumping between two sites, crossing a potential barrier under the effect of thermal activation [46].

#### 3.3.4. The Imaginary Spectrum and the Reality of Impedance

**Figure 11:**
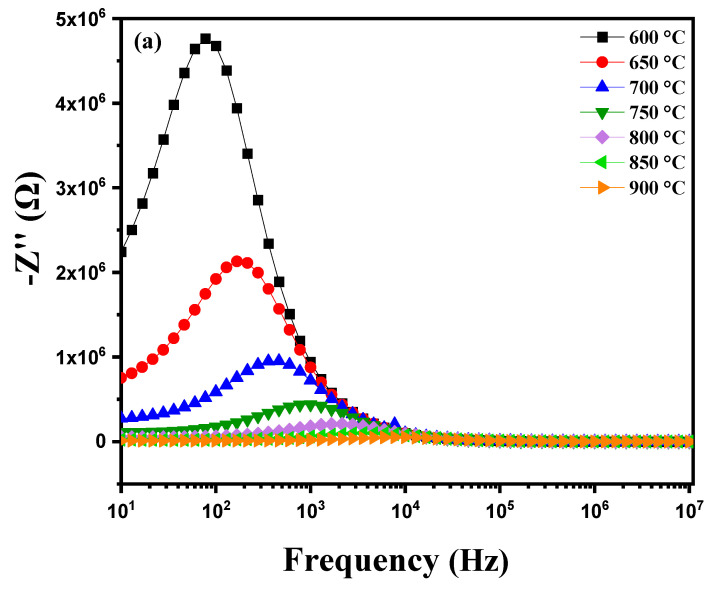

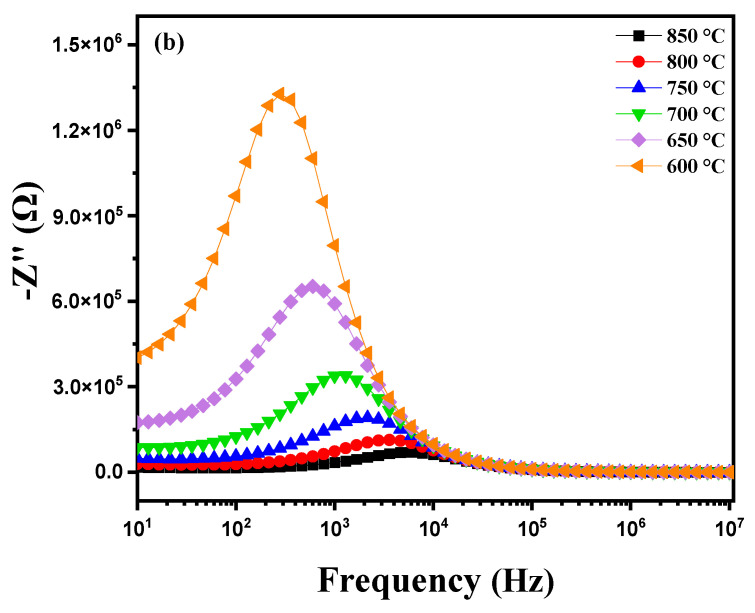
Diagram of the variation in the imaginary part of impedance (Z″) as a function of frequency: (**a**) heating curve from 600 °C to 900 °C and (**b**) cooling curve from 850 °C to 600 °C for modified marl.

Figure 11a shows the measurement of the imaginary part of impedance (Z″) as a function of frequency (ranging from 1 Hz to 1 MHz) at different temperatures, from 600 °C to 900 °C. Several important phenomena occur around the relaxation peak. At low temperatures, the relaxation frequency fmax is low, suggesting that the movement of charge carriers, such as vanadium oxides (VOs), requires significant time to transition from one site to another. This is due to reduced charge carrier mobility as the available thermal energy is insufficient to facilitate their movement. With increasing temperature, a decrease in the maximum value of Z″ is observed, while fmax shifts to higher frequencies. This shift to higher frequencies indicates that the relaxation time τ decreases as temperature rises. In other words, charge carriers become more mobile, reducing the time required for relaxation and increasing the frequency at which this relaxation occurs [48,49,50]. This behavior highlights the impact of temperature on charge carrier dynamics in the material, accelerating their mobility and thereby altering dielectric relaxation properties. When analyzing the imaginary part of impedance (Z″) of this modified marl pellet as a function of frequency (from 1 Hz to 1 MHz) at various temperatures (from 850 °C to 600 °C), Figure 11b reveals several characteristic phenomena, especially around impedance peaks observed during cooling. As temperature decreases, the peaks in the Z″ spectrum may shift toward lower frequencies. This shift to lower frequencies is often due to reduced ion and charge mobility within the material as temperature drops, altering relaxation and conduction characteristics. Furthermore, the temperature reduction in charge carriers (VO) can lead to an enlargement of peaks in the imaginary part of impedance. This is generally associated with an increase in the material’s relative permittivity influenced by changes in conduction and relaxation mechanisms at lower temperatures.

**Figure 12:**
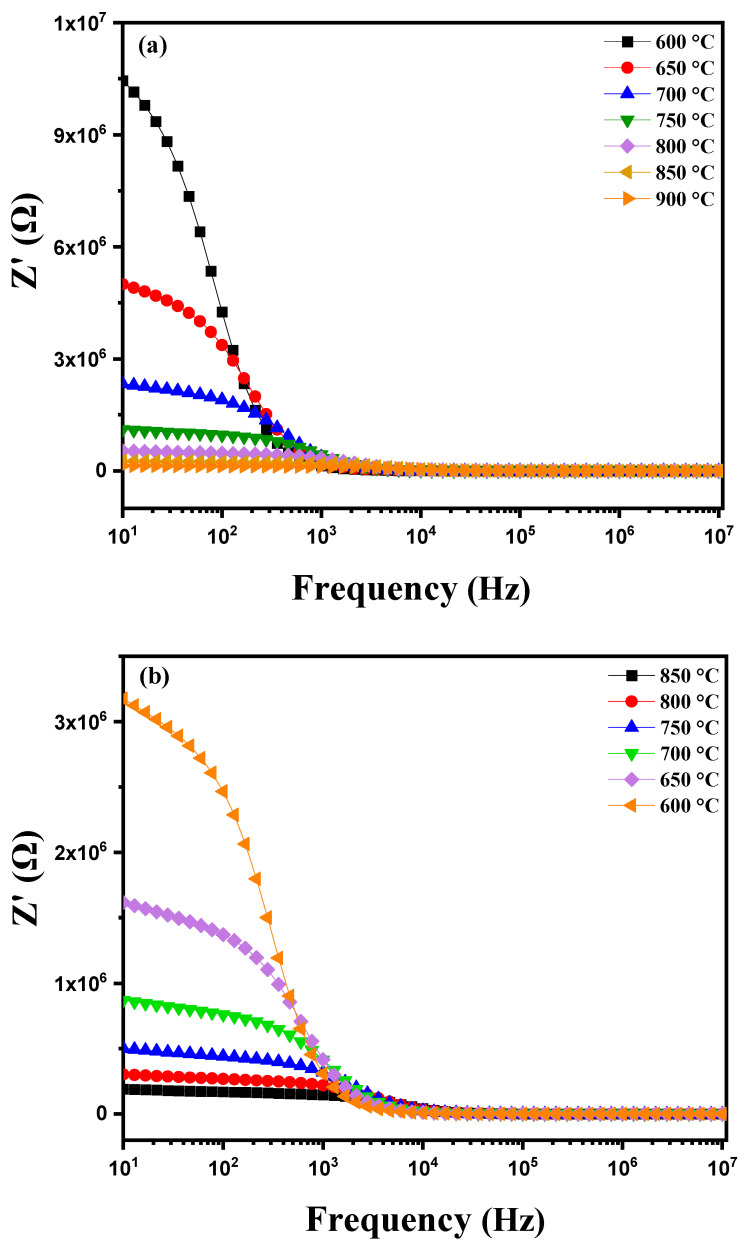
Diagram of the variation in the real part of impedance (Z′) as a function of frequency: (**a**) heating curve from 600 °C to 900 °C and (**b**) cooling curve from 850 °C to 600 °C for modified marl.

The variation in the real component of impedance (Z′) of a modified ceramic as a function of frequency and temperature (600 °C to 900 °C) is shown in Figure 12a, which displays distinctive features. As frequency increases, a gradual decrease in Z′ is observed approaching zero at high frequencies. This decrease is associated with the attenuation of polarization mechanisms such as electronic, ionic, orientational and interfacial polarization, which are more pronounced at low frequencies [51]. At these low frequencies, Z′ values are high due to the combined influence of these different polarizations. Conversely, at high frequencies the contribution from dipolar and interfacial mechanisms significantly decreases, resulting in the stabilization of Z′ at lower values [52]. Additionally, the decrease in Z′ with increasing frequency and temperature indicates an increase in alternating current (AC) conductivity. At low frequencies, a notable decrease in Z′ with increasing temperature reveals a behavior of resistance with a negative temperature coefficient (NTCR), meaning that the material’s resistance decreases as temperature rises, thereby enhancing overall conductivity [53,54]. When plotting the real part of impedance (Z′) of this modified marl pellet as a function of frequency (from 1 Hz to 1 MHz) during cooling at various temperatures (between 850 °C and 600 °C), Figure 12b shows an increase in Z′ as the temperature decreases. This behavior indicates a resistance phenomenon with a negative temperature coefficient (NTCR) commonly observed in semiconductors and certain ceramics. The NTCR reflects the fact that the material’s electrical resistance increases as temperature decreases. At high temperatures, the mobility of ions and charges in the material is greater, resulting in a decrease in resistance and consequently a reduction in the real part of impedance Z′. At lower temperatures, this mobility decreases, leading to an increase in resistance and thus an elevation in Z′. This behavior is typical of materials with a strong dielectric component or those undergoing phase transitions at high temperatures, thereby influencing the frequency response of Z′.

#### 3.3.5. Modulus Spectroscopy Study

The complex modulus analysis allows for the exploration of a material’s electrical properties using the complex permittivity M*, which is divided into the real modulus M′ and the imaginary modulus M″. The real modulus M′ represents a material’s ability to store energy as a function of frequency and temperature, typically increasing at low frequencies and high temperatures due to the thermal activation of charge carriers. On the other hand, the imaginary modulus M″ is associated with energy losses and relaxation mechanisms, appearing as peaks at specific frequencies that decrease at high frequencies, indicating a reduction in energy dissipation through the material [55].

M* = 1/ε * = j C_0_Z* = M′+ j M″

**Figure 13:**
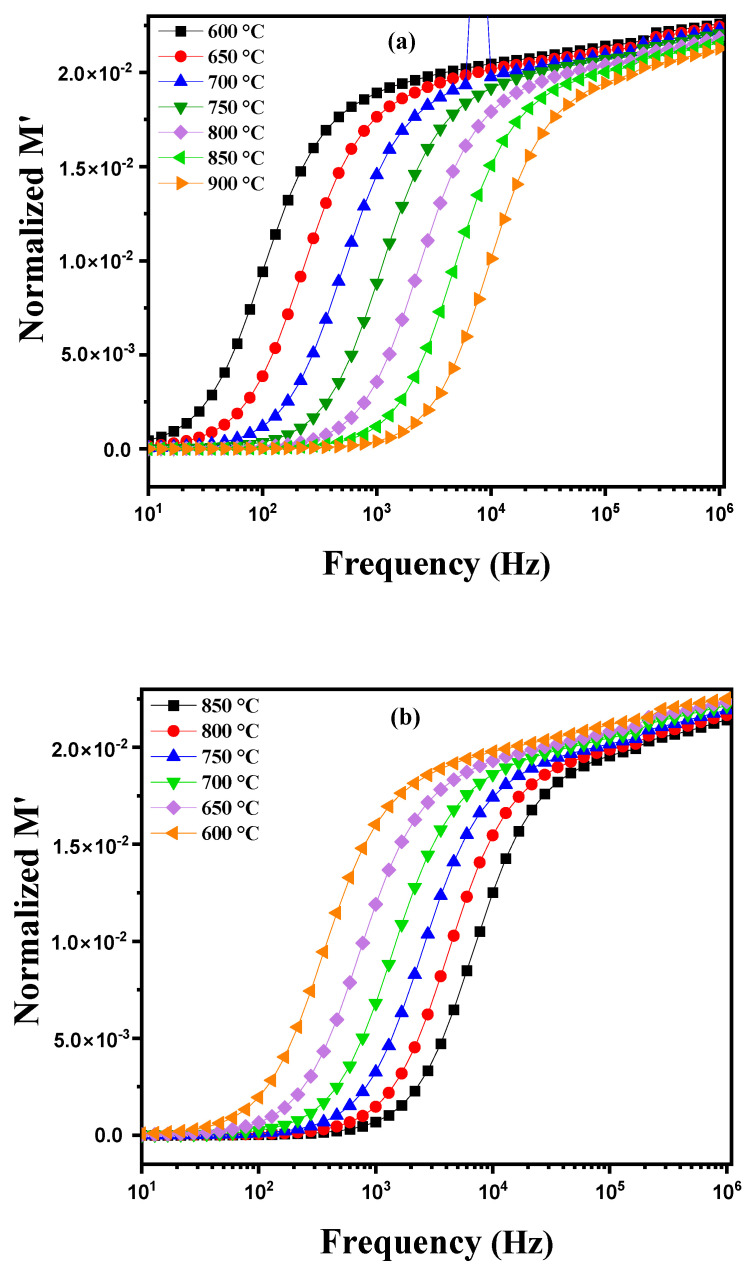
Real part of the modulus M′ as a function of frequency at different temperatures: (**a**) heating curve (600–900 °C) and (**b**) cooling curve (850–600 °C) for modified marl.

Nyquist plots, which plot M″ against M′, are often used to visualize and interpret conduction and polarization processes in materials. These plots allow for the distinction between grain and grain boundary contributions to the dielectric response, thus providing detailed information on relaxation and conduction mechanisms. This analysis is crucial for understanding how materials respond to alternating electric fields, offering essential insights into the underlying conduction and polarization mechanisms that influence material performance at different temperatures and frequencies [24].

$$M^{\prime }=\frac{\varepsilon^{\prime }}{{\varepsilon^{\prime }}^{2}+{\varepsilon^{\prime \prime }}^{2}};M^{\prime \prime }=\frac{\varepsilon^{\prime \prime }}{{\varepsilon^{\prime \prime }}^{2}+{\varepsilon^{\prime \prime }}^{2}}$$

where ε′ and ε″ represent the real and imaginary parts of the dielectric permittivity, respectively. Figure 13a illustrates the variation in the real part of the permittivity modulus (M′) as a function of frequency at different temperatures, ranging from 600 °C to 900 °C. Distinct behaviors are observed according to the temperature. At high frequencies, as the temperature increases, M′ tends to decrease and approaches zero. This phenomenon can be attributed to a reduction in the material’s real permittivity at high temperatures, possibly due to a decrease in the density of available charge carriers or changes in the material’s structure that affect its electrical properties. However, at lower frequencies, M′ shows a gradual upward trend. This increase is characteristic of the conduction process where available charge carriers move under the influence of the alternating electric field. As the frequency increases, the permittivity modulus increases until it reaches a saturation plateau after crossing a certain frequency threshold. This saturation results from the limit of the charge carriers’ response to rapid variations in the electric field, indicating that the charge carriers have reached their maximum capacity to contribute to the material’s conductivity at these high frequencies. Furthermore, a temperature rise improves the mobility of charge carriers, facilitating their movement and thereby increasing their contribution to conductivity. Consequently, at higher temperatures, the overall saturation level of M′ increases, reflecting an improved response of the material to alternating electric fields. This behavior demonstrates the complex interdependence between temperature, frequency, and the material’s electrical properties, highlighting the impact of these factors on the permittivity modulus [56,57,58]. Figure 13b shows the behavior of a modified diopside-based marl pellet during cooling, showing the evolution of the real part of the modulus M′ as a function of frequency as temperature decreases (from 850 °C to 600 °C). Several notable phenomena appear. As the temperature decreases, the mobility of charge carriers within the material is reduced. This reduction in mobility results in a decreased ability of charge carriers to respond to high-frequency excitations. Consequently, the saturation of M′, which directly depends on this mobility, also decreases at lower temperatures. Moreover, as the temperature drops, the M′ curve shifts toward lower frequencies. This shift indicates that the saturation threshold is reached at lower frequencies due to the reduced mobility of charge carriers. Thus, at lower temperatures the material requires lower frequencies to achieve M′ saturation, reflecting a reduced efficiency of the conduction process under these lower thermal conditions.

**Figure 14:**
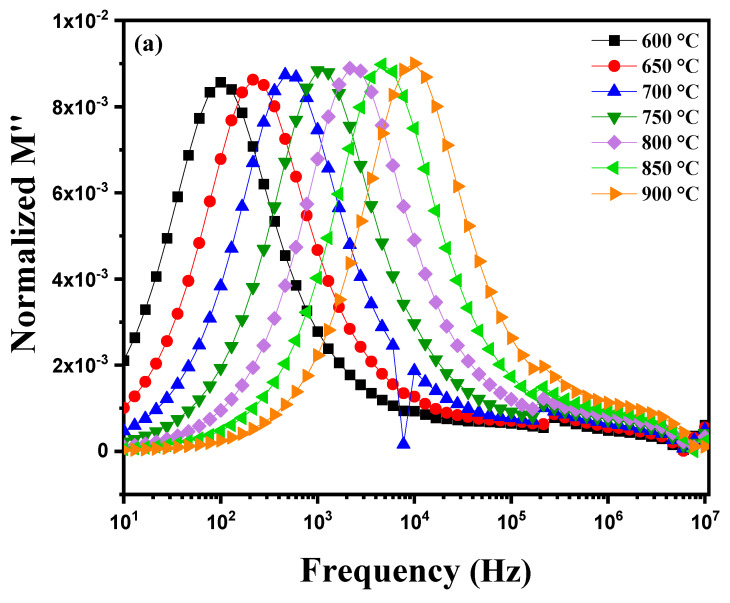

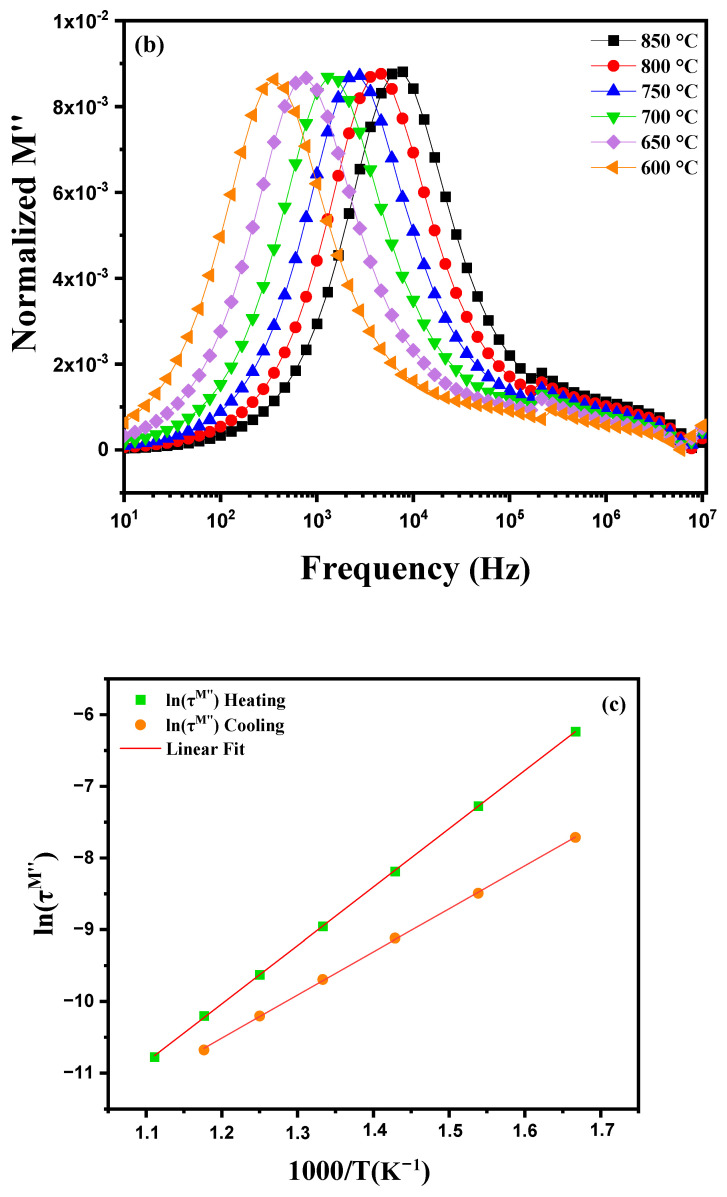
Imaginary part of the modulus M″ as a function of frequency at different temperatures: (**a**) heating curve (600–900 °C). (**b**) cooling curve (850–600 °C) and (**c**) plot of ln(στM″) as a function of the inverse temperature, 1000/T, for modified marl.

Figure 14a illustrates the variation in the imaginary part of the module M″ as a function of frequency at different temperatures ranging from 600 °C to 900 °C. Several phenomena can be observed for an electrically homogeneous pellet. With increasing temperature, the electrical and dielectric properties of the material undergo noticeable changes. A shift in the peaks of the imaginary module M″ towards higher frequencies is generally observed. This change is due to the acceleration of ionic movements and relaxation processes within the material as the temperature increases. Ions become more mobile and can respond more quickly to applied electric fields, leading to relaxation peaks at higher frequencies. The peaks observed in the M″ plots demonstrate relaxation behavior, favoring energy dissipation, as evidenced by the symmetrical displacements of the peaks. This behavior suggests that the sample exhibits a non-Debye relaxation mechanism [59]. Figure 14b shows the behavior of this pellet during cooling from 850 °C to 600 °C. A shift in the peaks toward lower frequencies is observed. For an electrically homogeneous pellet, this phenomenon can be attributed to the evolution of dielectric relaxation mechanisms and the dynamics of ionic conduction within the material. At high temperatures (near 850 °C), ions in the crystalline structure of diopside have reduced mobility compared to lower temperatures, causing the M″ peaks to appear at higher frequencies. As the pellet cools, ion mobility increases, allowing the ions to more effectively follow variations in the applied electric field. This increase in ionic mobility results in a shift in the M″ peaks toward lower frequencies. This shift indicates that relaxation processes become increasingly efficient at lower temperatures as ions can move more freely within the material’s structure. Figure 14c shows the variation in the logarithm of electrical conductivity, ln(στ), as a function of the inverse temperature 1000/T for two data series: one corresponding to heating, and the other to cooling. The linear fit is based on the Arrhenius relationship, which describes the behavior of a thermally activated process. During heating, charge carriers benefit from greater thermal energy, which enhances their mobility through the material’s crystalline grains. This leads to higher conductivity (less negative ln(στ) values) and shorter relaxation times as temperature increases. The linear behavior on the curve corresponds to thermal activation of processes within the material, with a steeper slope for heating reflecting a higher activation energy. During cooling, the behavior differs, indicating thermal hysteresis. Grains and especially grain boundaries play a more significant role. At low temperatures, charge carriers are trapped by defects located at the grain boundaries, making their mobility more difficult. This results in lower conductivity (more negative ln(στ) values) and longer relaxation times. The slope of the cooling curve is shallower, indicating that charge carriers are more affected by barriers at the grain boundaries.

**Figure 15:**
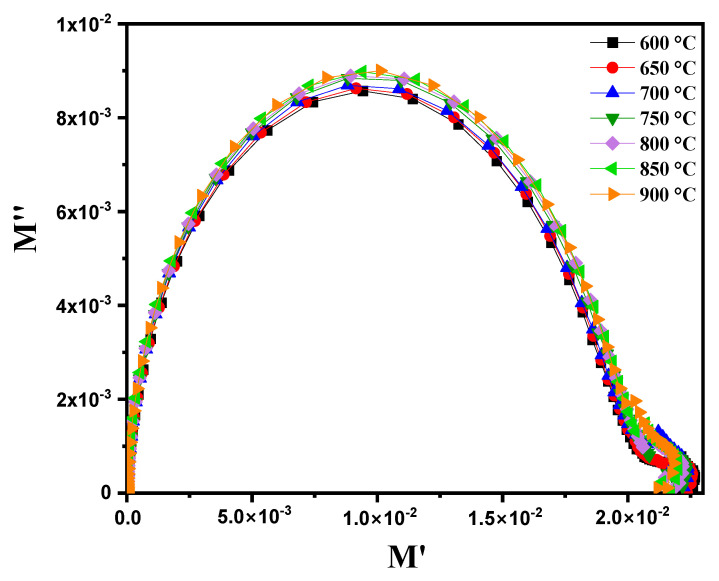
Complex modulus diagram (M″ vs. M’) for modified marl at different temperatures (600–900 °C).

When representing the variation in M″ (the imaginary part of the complex permittivity) as a function of M′ (the real part of the complex permittivity) at different temperatures ranging from 600 °C to 900 °C, Figure 15 highlights the appearance of several semicircles on the Nyquist diagram. This observation indicates significant changes in the dielectric response of the material as a function of the temperature. The semicircles are clearly separated in this compound. These results confirm the equivalent circuit presented in Figure 9c, which is not visible in the Nyquist impedance diagram for these materials [60].

**Figure 16:**
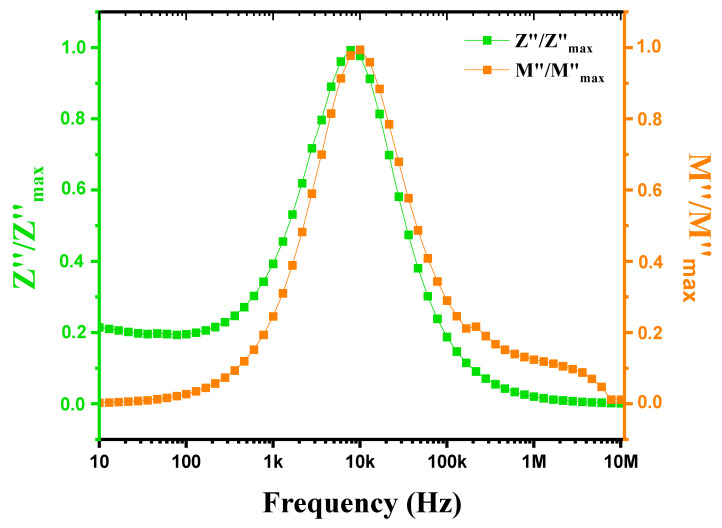
Variation in Z″/Z″max and M″/M″max with frequency for modified marl at 900 °C.

Figure 16 shows the evolution of the imaginary part of the normalized impedance (Z″/Z″max) and the normalized electrical modulus (M″/M″max) as a function of frequency, covering a range from 1 Hz to 1 MHz at a temperature of 900 °C. Characteristics indicative of the material’s dielectric properties are observed. The imaginary part of the impedance (Z″) reflects energy losses due to relaxation and conduction processes, showing peaks at specific frequencies where these losses are maximal, often linked to specific relaxations or conduction transitions. Similarly, the normalized electrical modulus (M″) reveals frequencies of maximum losses, reflecting phenomena similar to those observed in Z″. When the peaks of Z″/Z″max and M″/M″max are very close, it indicates that the relaxation processes influencing dielectric and impedance losses are similar, suggesting a strong interaction between polarization and conduction phenomena. This peak proximity may be due to relaxations that similarly affect both the imaginary part of the impedance and the electrical modulus, indicating that these phenomena occur at close frequencies. Closely spaced peaks in Z″/Z″max and M″/M″max at 900 °C reflect common relaxation and conduction processes, highlighting the complexity of the material’s electromagnetic properties at high temperatures and suggesting the coexistence of localized and extended relaxations [61]. This absence of a superposition of the Z″ and M″ peaks again suggests a deviation from the ideal Debye-type behavior in our ceramics [62].

## 4. Conclusions

The modification of marl by the incorporation of diopside has made it possible to considerably improve its dielectric properties, in particular by improving the dielectric constant and the dielectric losses. These improvements make this material more efficient for applications requiring optimized storage and transmission of electrical charges, in particular in capacitors, electrical insulators, and substrates for printed circuits. Compared to traditional dielectric materials such as BaTiO_3_ (barium titanate) or Al_2_O_3_ (alumina), marl modified with diopside has the advantage of being derived from an abundant and potentially more economical natural resource while offering interesting performance in terms of minimizing energy losses.

The obtained results confirm that this modified material is promising for advanced electronic applications. However, a more in-depth comparative analysis with industrial dielectric ceramics is needed to better position the modified marl in this field. For example, although BaTiO_3_ has a high permittivity, it is expensive and requires complex synthesis processes. On the other hand, diopside-modified marl, while having a slightly lower permittivity, could be a viable alternative due to its availability and reduced cost. The main objective of this study, which aimed to develop a more economical and efficient material for electronic applications, has therefore been partially achieved, paving the way for further research to further optimize its properties and explore new applications.

## Figures and Tables

**Table 1:** XRF analysis results of the elemental and oxide composition of raw marl.

Element/Oxide	Element [%]	Oxide [%]
Na/Na_2_O	3.64	2.78
Mg/MgO	1.88	1.76
Al/Al_2_O_3_	10.96	12.54
Si/SiO_2_	59.74	68.12
P/P_2_O_5_	0.148	0.169
S/SO_3_	0.0755	0.0928
K/K_2_O	3.18	1.85
Ca/CaO	7.67	5.1
Ti/TiO_2_	0.754	0.584
V/V_2_O_5_	0.0442	0.0367
Cr/Cr_2_O_3_	0.0132	0.0088
Mn/MnO	0.0735	0.0433
Fe/Fe_2_O_3_	6.98	4.54
Zn/ZnO	0.0233	0.0126
As/As_2_O_3_	0.296	0.149
Rb/Rb_2_O	0.027	0.0168
Sr/SrO	0.0479	0.0213
Zr/ZrO_2_	0.0504	0.0264
Nb/Nb_2_O_5_	/	0.0046
Ba/BaO	0.076	0.0387
Cl	4.25	2.08
Sum	99.929	99.974

**Table 2:** Electrical parameters of the equivalent circuit obtained from the complex impedance spectrum of the modified marl.

T(°C)	Rgb (Ω)	Cgb (F)	Qgb (F.sα1)	A	Rg (Ω)	Cg (F)	Qg (F.sα1)	β (°)
600 °C	5.974 × 10^6^	1.004 × 10^−9^	0.169 × 10^−9^	0.1782	3.238 × 10^6^	1.559 × 10^−9^	2.596 × 10^−9^	32.57
650 °C	2.975 × 10^6^	0.1925 × 10^−9^	0.2776 × 10^−9^	0.1817	1.575 × 10^6^	1.671 × 10^−9^	3.136 × 10^−9^	36.14
700 °C	1.359 × 10^6^	0.1811 × 10^−9^	0.3606 × 10^−9^	0.1751	8.24606 × 10^5^	1.81 4 × 10^−9^	3.826 × 10^−9^	33.25
750 °C	7.10739 × 10^5^	0.1784 × 10^−9^	0.397 × 10^−9^	0.2573	6.600315 × 10^5^	1.964 × 10^−9^	4.266 × 10^−9^	30.45
800 °C	4.15429 × 10^5^	0.1679 × 10^−9^	0.4256 × 10^−9^	0.1454	3.6902 × 10^5^	2.175 × 10^−9^	4.976 × 10^−9^	38.62
850 °C	2.30314 × 10^5^	0.1479 × 10^−9^	0.5643 × 10^−9^	0.3565	1.77127 × 10^5^	2.419 × 10^−9^	5.1656 × 10^−9^	34.05
900 °C	1.516 × 10^5^	0.1388 × 10^−9^	0.669 × 10^−9^	0.3575	9.5970 × 10^4^	2.675 × 10^−9^	6.163 × 10^−9^	32.38
850 °C	6.439 × 10^5^	0.1609 × 10^−9^	0.6365 × 10^−9^	0.1594	1.99689 × 10^5^	2.714 × 10^−9^	6.103 × 10^−9^	34.88
800 °C	9.491 × 10^5^	0.1823 × 10^−9^	0.5312 × 10^−9^	0.1711	3.19745 × 10^5^	2.516 × 10^−9^	5.393 × 10^−9^	37.47
750 °C	1.612 × 10^6^	0.1974 × 10^−9^	0.4025 × 10^−9^	0.2013	5.68456 × 10^5^	3.227 × 10^−9^	4.553 × 10^−9^	31.59
700 °C	2.684 × 10^6^	1.164 × 10^−9^	0.3229 × 10^−9^	0.2288	9.98857 × 10^5^	4.619 × 10^−9^	4.193 × 10^−9^	30.25
650 °C	5.011 × 10^6^	1.364 × 10^−9^	0.227 × 10^−9^	0.2333	1.747 × 10^6^	6.033 × 10^−9^	3.733 × 10^−9^	30.15
600 °C	8.562 × 10^6^	1.564 × 10^−9^	0.2957 × 10^−9^	0.2394	3.775 × 10^6^	8.049 × 10^−9^	2.803 × 10^−9^	30.64

## Data Availability

Data is contained within the article.
